# The Ability of Different Ketohexoses to Alter Apo-A-I Structure and Function In Vitro and to Induce Hepatosteatosis, Oxidative Stress, and Impaired Plasma Lipid Profile in Hyperlipidemic Zebrafish

**DOI:** 10.1155/2018/3124364

**Published:** 2018-05-21

**Authors:** Dhananjay Yadav, Suk-Jeong Kim, Myung Ae Bae, Jae-Ryong Kim, Kyung-Hyun Cho

**Affiliations:** ^1^Department of Medical Biotechnology, Yeungnam University, Gyeongsan 38541, Republic of Korea; ^2^Drug Discovery Platform Technology Team, Korea Research Institute of Chemical Technology, Daejeon 305-343, Republic of Korea; ^3^Department of Biochemistry and Molecular Biology, Smart-Aging Convergence Research Center, College of Medicine, Yeungnam University, Daegu 705-717, Republic of Korea

## Abstract

In the current study, we have tested the nonenzymatic glycation activities of ketohexoses, such as tagatose and psicose. Although tagatose-treated apoA-I (t-A-I) and psicose-treated apoA-I (p-A-I) exerted more inhibitory activity you cupric ion-mediated low-density lipoprotein (LDL) oxidation and oxidized LDL (oxLDL) phagocytosis into macrophage than fructose-treated apoA-I (f-A-I). In the lipid-free state, t-A-I and f-A-I showed more multimerized band without crosslinking. Since t-A-I lost its phospholipid binding ability, the rHDL formation was not as successful as f-A-I. However, injecting t-A-I showed more antioxidant activities in zebrafish embryo under the presence of oxLDL. Three weeks of consumption of fructose (50% of wt in Tetrabit/4% cholesterol) showed a 14% elevation of serum triacylglycerol (TG), while tagatose-administered group showed 30% reduction in serum TG compared to high cholesterol control. Fructose-fed group showed the biggest area of Oil Red O staining with the intensity as strong as the HCD control. However, tagatose-consumed group showed much lesser Oil Red O-stained area with the reduction of lipid accumulation. In conclusion, although tagatose treatment caused modification of apoA-I, the functional loss was not as much severe as the fructose treatment in macrophage cell model, zebrafish embryo, and hypercholesterolemic zebrafish model.

## 1. Introduction

It has been widely accepted that glycation is a major process that degenerates protein function and structure, which is a direct outcome of chronic metabolic diseases, such as diabetes [1], atherosclerosis [2], and aging [3]. Fructose treatment can cause glycation up to 10 times higher with the production of advanced glycated end (AGE) products than that of glucose, and higher fructose consumption promotes triglyceride synthesis [4, 5]. It has been known that major target of glycation via Maillard reaction is serum hemoglobin (Hgb), glycated Hgb level has been used as a diagnostic marker of diabetes [6]. Previous studies have concluded that glycation could occurs in high-density lipoproteins (HDL) and apolipoproteins in blood [7, 8].

High-density lipoprotein-cholesterol (HDL-C) is inversely associated with the incidence of cardiovascular disease [9] and is directly related to longevity [10]. HDL has antioxidant and anti-inflammatory potential [11]. Apolipoprotein A-I is the principal protein of HDL, exerting to suppress atherosclerosis [12]. Our study group have reported that fructose-mediated apoA-I glycation results in the acute loss of the beneficial functions of apoA-I and HDL with respect to its antisenescence, antioxidant, and antiatherosclerosis activities [8, 13, 14] and that could be suggested due to the oligomerization (crosslinking of monomeric apoA-I to form dimers, trimers, tetramers, etc.). It is a process of multimerization which can contribute to amyloid production and impairment of lipoprotein functionality. The functionality and structural modifications coupled with increased protein degradation lead to severe health disorders [2, 3].

As fructose is a ketohexose family, there might be a possibility that other ketohexose can cause similar glycation process and physiological effects. Among ketohexoses, D-tagatose is the epimer of d-fructose differing only in the positioning of hydroxyl group on the 4th carbon. Tagatose has grabbed attention as a potent candidate of antidiabetic agents [15] and has been established as the safe sugar (GRAS) by World Health Organization (WHO) for use in food and beverages. It has been reported that dietary supplementation of tagatose in type 2 diabetes leads to weight loss and raises the HDL-C levels [16]. Unfortunately, this study was not placebo-controlled.

Not only d-tagatose, d-psicose, which is a C-3 epimer of d-fructose, has lesser sweetness than sucrose with no calories, rather exhibits hypoglycemic, hypolipidemic, and antioxidant activities [17–20]. The supplementation of d-psicose in the diet of male rats suppressed the hepatic fatty acid synthase and glucose 6-phosphate dehydrogenase enzymes and thereby reduces adipose tissue weight [19]. These properties make it a more promising agent for ameliorating diabetes and its related conditions [21]. However, the explanation for the beneficial effect of tagatose and psicose has not yet been deciphered, especially in serum proteins regarding glycation and its physiological mechanism. Since there has been no report about the potential effect of tagatose and psicose in lipoprotein metabolism and there is a possibility that both of them can affect serum protein via nonenzymatic glycation process, hence we tested the putative effect of tagatose treatment as well as fructose and psicose.

## 2. Materials and Methods

### 2.1. Materials

Cholesterol (# C-3045) was purchased from Sigma (St. Louis, MO, USA). d-tagatose (FW 180.16, cat #T1501), d-fructose (FW180.16, cat # F0060), and d-psicose (FW 180.16, cat # P1699) were purchased from Tokyo Chemical Industry (Tokyo, Japan).

### 2.2. Purification of apoA-I

Human plasma was used to purify ApoA-I using several techniques such as ultracentrifugation, column chromatography, and organic solvent extraction method described by Brewer et al. [22]. The purified apoA-I was lyophilized at −80°C until use.

### 2.3. Treatment of Ketohexose to apoA-I

Lipid-free apoA-I (10 mg/mL) in its processed state was kept in 200 mM potassium phosphate/0.02% sodium azide buffer (pH 7.4) supplemented with each of the ketohexose (final 250 mM) for up to 90 hrs in an incubator with 5% CO_2_ at 37°C. The measure of advanced glycation was performed spectrophotometrically using fluorescence at 440 nm (emission) and 370 nm (excitation), the procedure was however slightly modified [23].

### 2.4. Synthesis of Reconstituted HDL and Analysis

Table 1 indicates the synthesis and characterization of rHDL containing each ketohexose. Discoidal rHDL was prepared by the sodium cholate dialysis method [24] using initial molar ratios of palmitoyl oleoyl phosphatidylcholine (POPC) : cholesterol : apoA-I : sodium cholate of 95 : 5 : 1 : 150. As the rHDL particles employed in the procedure were pure enough and exhibited extreme uniformity, further processing was deemed unnecessary. PAGGE or native polyacrylamide gradient gel electrophoresis was carried out in order to determine the size of the rHDL particles and to compare them with standard globular proteins (Cat# 17-0445-01 Amersham Pharmacia, Uppsala, Sweden). The Pharmacia Phast system was obtained from GE Healthcare, Uppsala, Sweden. Gel Doc® XR (Bio-Rad, Hercules, CA, USA) complemented with Quantity One software, version 4.5.2 was employed to compare the relative movement of particles, while its content of protein was measured by Lowry method but improvised by as modified Markwell et al. [25] with standard as BSA.

### 2.5. Circular Dichroism and Fluorospectroscopy

The assaying technique of circular dichroism (CD) spectroscopy (J-715 Spectropolarimeter Jasco, Tokyo, Japan) was used to unravel the quantity of alpha-helices prevalent in the free and bound protein-lipid interaction states. The circular light absorption pattern was obtained from 250–190 nm at 25°C, 0.1 cm was path length, 1.0 nm was bandwidth, speed was 50 nm/min, and a 4 sec response time. The purified protein specimens were made fructose free by dialysis against TBS, self-ligation was averted by dilution of the lipid-free apolipoproteins [26] to 0.07 mg/mL and to 0.1 mg/mL of the lipid-bound apolipoprotein. Four scans were obtained and averaged.

The analysis of molar ellipticity at 222 nm revealed the content of alpha-helices. Circular dichroism spectra with ketohexose-treated apoA-I in lipid-free and lipid-bound are represented in Supplementary Figure 2.

The perusal of the wavelengths of fluorescence (WMF) particularly of the Trp residues was done by a LS55 spectrofluorometer (Perkin-Elmer, Norwalk, CT, USA) using WinLab software package 4.00 (Perkin-Elmer). The excitation wavelength was chosen to be 295 nm in order to steer clear of the interference from tyrosine fluorescence. Emissions were categorically checked from 305–400 nm.

### 2.6. Purification of Low-Density Lipoprotein (LDL) and Its Oxidation

LDL (1.019 < d < 1.063) was obtained from human plasma and purified by ultracentrifugation, the density was adjusted by adding NaCl, followed by centrifugation at 100,000*g* for 22 hours at 10°C temperature (Himac CP-90*α* Hitachi, Tokyo, Japan). The oxidized LDL (oxLDL) was procured postincubation with CuSO_4_ (final concentration, 10 *μ*M) for 4 hr at 37°C. Subsequently, it was filtered (0.2 *μ*m) and investigated by using a thiobarbituric acid reacting substances (TBARS) assay to establish the degree of oxidation [27].

### 2.7. Cell Culture

The human monocyte cell line, THP-1, was acquired from the (ATCC, #TIB-202™; Manassas, VA, USA) and sustained in RPMI1640 (Hyclone, Logan, Utah) with 10% fetal bovine serum (FBS) supplemented. The cell line below 20 passages were used and incubated in phorbol 12-myristate 13-acetate (PMA; final 150 nM) supplemented medium in 24-well plates for 48 hours at 37°C in a humidified incubator (5% CO_2_) to induce macrophage differentiation.

### 2.8. LDL-Phagocytosis Assay

The differentiated and the adhered macrophages were coincubated with 400 *μ*L of fresh RPMI1640 medium supplemented with 1% FBS, 50 *μ*L of oxLDL [1 mg of protein/mL in phosphate-buffered saline (PBS)], and 50 *μ*L of each rHDL (1 mg/mL) for 48 hr at 37°C in a humidified incubator to test antiatherosclerotic activity [14]. Subsequently, the cells were washed thrice with PBS and then fixed in 4% paraformaldehyde for 10 minutes. Then the fixed specimens were further rinsed with 100% polypropylene glycol, then stained with Oil Red O staining solution (0.67%), and finally washed with distilled water. THP-1 macrophage-derived foam cells were then imaged and photographed using a Nikon Eclipse TE2000 microscope (Tokyo, Japan) at 600x magnification.

### 2.9. Western Blot

After 48 hr incubation, the harvested cell was lysed by treatment of RIPA buffer (Radioimmunoprecipitation assay buffer, 150 mM NaCl, 1% NP-40, 0.5% sodium deoxycholate, 0.1% SDS, 50 mM Tris (pH 8.0)). Cell lysates were analyzed by western blot analysis using antihuman apoA-I antibody (Ab7613; Abcam, Cambridge, UK) and GAPDH (Ab8229, Abcam), antitumor necrosis factor (TNF)-*α* (SC52746, Santa Cruz, CA, USA). Protein content from each lysate was measured using Bradford assay (Bio-Rad, Hercules, CA, USA) before loading equal amounts of protein (25 *μ*g/lane) into 13% SDS-PAGE gels.

### 2.10. Zebrafish

Wild-type zebrafish and their embryos were sustained as per the standard protocols [28] permitted by the Committee of Animal Care and Use of Yeungnam University (Gyeongsan, Korea). The zebrafish, larvae, and embryos were maintained in a system cage and 6-well plates at 28°C during exposure to the 14 : 10 hrs light : dark cycle.

### 2.11. Microinjection of Zebrafish Embryos

The Pneumatic PicoPump of the make PV820; World Precision Instruments, Sarasota, FL, USA was used for microinjecting the day one of fertilized embryo or 1 day postfertilization (dpf). This PV280 also had a magnetic manipulator (MM33; Kantec, Bensenville, IL, USA) fitted with a pulled microcapillary pipette-using device (PC-10; Narishige, Tokyo, Japan). The oxLDL (13 ng of protein) and ketohexose-treated apoA-I (50 ng of protein) were coinjected in total 100 nL volume as our previous report [14]. Following injection, live embryos were observed under a stereomicroscope (Motic SMZ 168; Hong Kong) and photographed using a Motic cam2300 CCD camera.

### 2.12. Imaging of Reactive Oxygen Species (ROS)

After treatment with oxLDL in the presence of apoA-I, the increased ROS measures the extent of general oxidative stress were seen using dihydroethidium (DHE, cat # 37291; Sigma, St. Louis, MO) [29]. The image of the embryo stage was procured through fluorescence observation (Ex = 588 nm and Em = 605 nm) using a Nikon Eclipse TE2000 microscope (Tokyo, Japan). The measurement of fluorescent area in the embryo was done by a computer-guided morphometry using the Image Proplus software (version 4.5.1.22; Media Cybernetics, Bethesda, MD, USA).

### 2.13. In Vivo Test Using Hypercholesterolemic Zebrafish

The AB stain of Zebrafish was locally purchased. The preservation of zebrafish and procedures were permitted by the Committee of Animal Care and Use of Yeungnam University (Gyeongsan, Korea).

Tetrabit powder (47.5% crude protein, 6.5% crude fat, 2.0% crude fiber, 10.5% crude ash, vitamin A [29,770 IU/kg], vitamin D_3_ [1860 IU/kg], vitamin E [200 mg/kg], and vitamin C [137 mg/kg]; Tetrabit Gmbh D49304, Melle, Germany) was mixed with fructose or tagatose (final concentrations, 50% in tetrabit [wt/wt]). The mixture was lyophilized post its complete dissolution in water, the remainder was further ground into a fine powder. The tetrabit powder in isolation and also mixed with the ketohexose was blended with diethyl ether solution of cholesterol to create a 4% cholesterol. We described the preparation of food diet of zebrafish in our previous report containing 4% cholesterol (final concentration) high cholesterol diet (HCD) [30]. The same procedure was used to prepare mixture of the normal diet (ND) to remove the possibility of artifact caused by the solvent (diethyl ether).

The groups (*n* = 70) were fed with the assigned diet (10 mg/day/fish) without any exception; the details have been shown in Table 2. The zebrafish were kept at 28 ± 1°C under a 14 : 10 hrs light : dark cycle.

### 2.14. Blood Analysis

After consumption of high cholesterol diet and ketohexose for 3 weeks, blood was aspirated from the hearts of the fish and mixed with 5 *μ*L of 1 mM PBS-ethylenediaminetetraacetic acid (EDTA) and was subsequently transferred in EDTA-treated tubes. The plasma (30–40 *μ*L) was separated by centrifugation from 10 zebrafish samples. Plasma total cholesterol (TC) and triglycerides (TG) were measured by using commercial assay kits (Wako Pure Chemical, Osaka, Japan). The concentration of glutamic oxaloacetic transaminase (GOT) was determined using a commercially available assay kit (Asan Pharmaceutical, Hwasung, Korea).

### 2.15. Plasma Cholesteryl Ester Transfer Protein (CETP) Activity

The cholesteryl ester- (CE-) donor consisting of apolipoprotein A-I (apoA-I) and cholesteryl oleate, reconstituted HDL (rHDL), was produced [31] with trace quantities of [^3^H]-cholesteryl oleate (TRK886, 3.5 *μ*Ci/mg of apoA-I; GE Healthcare).

The CE-transfer reaction occurred in a 300 *μ*L reaction mixture comprising of uniformly diluted zebrafish plasma (50 *μ*L) as a cholesteryl ester transfer protein (CETP) source. The [^3^H]-CE-rHDL (50 *μ*L, 0.25 mg/mL) and human LDL (50 *μ*L, 0.25 mg/mL) were used as cholesteryl (CE)-donor and CE-acceptor, in that order. Postincubation at 37°C, this reaction was stopped through centrifugation at 10,000*g* for 3 minutes at 4°C. The supernatant containing CE-acceptor (150 *μ*L) was taken for to scintillation counting, and the percentage transfer of [^3^H]-CE from rHDL to LDL was calculated.

### 2.16. Histologic Analysis

Briefly, after the zebrafish were sacrificed, the liver was fixed with 4% paraformaldehyde for 24 hr. The stained liver samples were subsequently entrenched in Tissue-Tek OCT compound (Thermo, Walldorf, Germany) and frozen. Further, 7 *μ*m sections of these tissues were mounted on 3-APS (3-aminopropyltriethoxysilane) coated slides and viewed under a Leica microcryotome (model CM1510s, Heidelberg, Germany). Seven successive sectioned slides of each zebrafish were first stained with Oil Red O and then counterstained with hematoxylin which highlighted the fatty streak lesions. To compare the extent of oxidative stress in these tissues, the totality of reactive oxygen species (ROS) was seen with dihydroethidium (DHE, cat # 37291; Sigma, St. Louis, MO) [14, 26] postmicrotome sectioning by using a Nikon Eclipse TE2000 microscope (Tokyo, Japan). Section fluorescence was measured through a computer-aided morphometry using Image Proplus software (version 4.5.1.22; Media Cybernetics, Bethesda, MD, USA).

### 2.17. Statistical Analysis

Resultant output were statistically analyzed as the mean±SD obtained from three independent experimental repetitions. Comparisons between results was made by Students *t*-test and one-way ANOVA (Bonferroni *t*-test) using SPSS program (version 12.0; SPSS Inc., Chicago, IL, USA). The values were tested for significance at *P* < 0.001, *P* < 0.05.

## 3. Results

### 3.1. Glycation by Ketohexose Treatment

**Figure 1:**
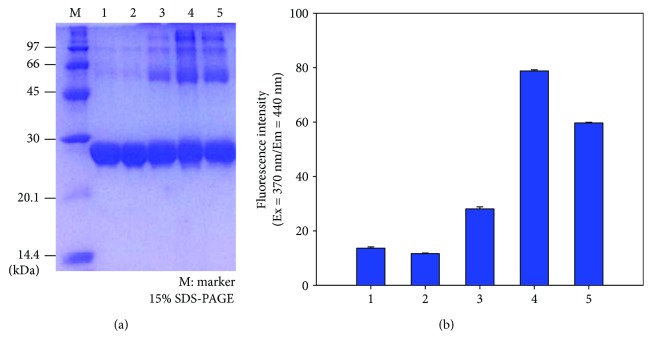
Electrophoretic characterization of modified apoA-I by each ketohexose. (a) Electrophoretic patterns of the glycated apoA-I in the lipid-free state on native gel (8–25% native gradient gel electrophoresis. Lane 1: native A-I, Lane 2: H2O-A-I, Lane 3: f-A-I, Lane 4: t-A-I, and Lane 5: p-A-I. (b) Glycation extent of apoA-I by each ketohexose treatment (Lane 1: native A-I, Lane 2: H2O-A-I, Lane 3: f-A-I, Lane 4: t-A-I, and Lane 5: p-A-I).

Under high dosage of ketohexose (final 50 mM) treatment in the lipid-free state, without crosslinking reaction, all ketohexose-treated apoA-I showed more multimerized pattern than control (H_2_O-treated apoA-I) as shown in Figure 1(a). After 72 hr incubation, tagatose-treated apoA-I (t-A-I) showed the strongest multimerization pattern from SDS-PAGE (Figure 1(a)) with the highest yellowish fluorescence (Figure 1(b)) from the Maillard reaction. Psicose treatment showed 2nd strongest multimerization and fluorescence.

Native polyacrylamide gel electrophoresis without sample boiling revealed that the ketohexose-treated apoA-I showed different band distribution and mobility in the lipid-free state as shown in Supplementary Figure 1A. They had an additional band around 88–89 Å and faster mobility in lower band position around 58–60 Å, while H_2_O-treated apoA-I or native apoA-I showed different electromobility with a major band around 71 Å. In the lipid-bound state, as shown in Supplementary Figure 1B, ketohexose-treated apoA-I showed decreased particle size around 93–95 Å, while H_2_O-treated apoA-I showed distinct two bands around 95 and 109 Å. t-A-I-rHDL showed the weakest band intensity and the more band in the bottom, lipid-free apoA-I.

### 3.2. Phospholipid-Binding Ability

**Figure 2:**
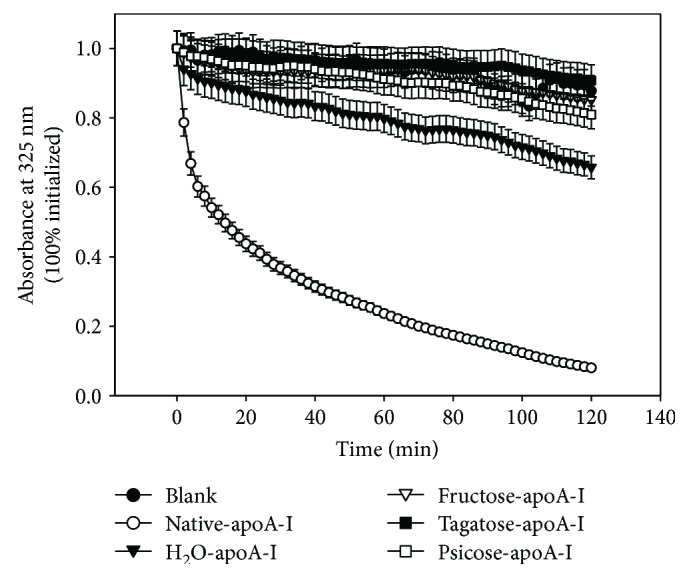
Phospholipid-binding ability of the ketohexose-treated apoA-I measured at an absorbance of 325 nm.

Up to 120 min incubation with DMPC (dimyristoyl phosphatidylcholine), t-A-I showed almost loss of phospholipid-binding ability, while f-A-I and p-A-I also showed impairment of binding ability (Figure 2). H_2_O-treated apoA-I showed the fastest phospholipid-binding ability with half time for clearance (T_1/2_ = 14 min).

### 3.3. Inhibition of Cupric Ion-Mediated LDL Oxidation

During 120 min incubation, cupric ion-treated LDL showed the highest elevation of absorbance at 234 nm (A_234_). There was no notable difference of A_234_ between ketohexose-treated apoA-I in the lipid-bound state.

**Figure 3:**
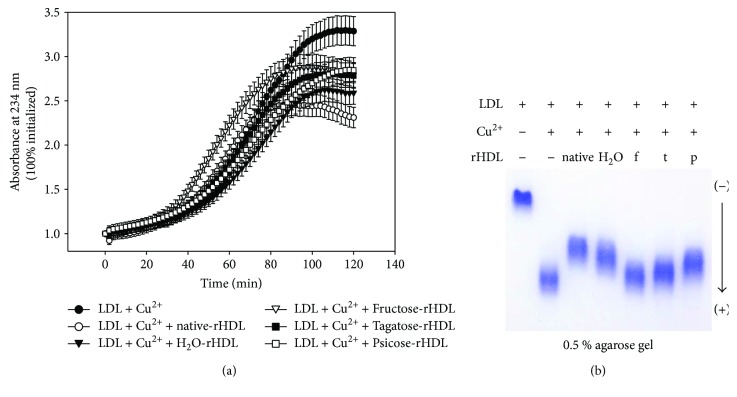
Antioxidant ability of apoA-I, which was treated by each ketohexose, against oxidation of LDL. (a) Detection of conjugated diene midst copper-mediated LDL oxidation. Continuous monitoring of conjugate diene level at absorbance 234 nm (A_234_) during copper-mediated oxidation in the presence of either rHDL containing native apoA-I or ketohexose treated apoA-I. (b) Electromobility of LDL, which was oxidized by Cu^2+^ under presence of ketohexose treated apoA-I-rHDL, on 0.5% agarose gel.

Although t-A-I-treated LDL showed more oxidized product than that of n-A-I-treated LDL, t-A-I exhibited better antioxidant activity than f-A-I from monitoring of conjugated diene (A_234_) as shown in Figure 3. Agarose electrophoresis also showed that f-A-I-treated LDL revealed the fastest electromobility, indicating the extent of oxidation. t-A-I- and p-A-I-treated LDL showed slower electromobility than f-A-I, suggesting their superior antioxidant ability than f-A-I.

### 3.4. Uptake of oxLDL into Macrophage

**Figure 4:**
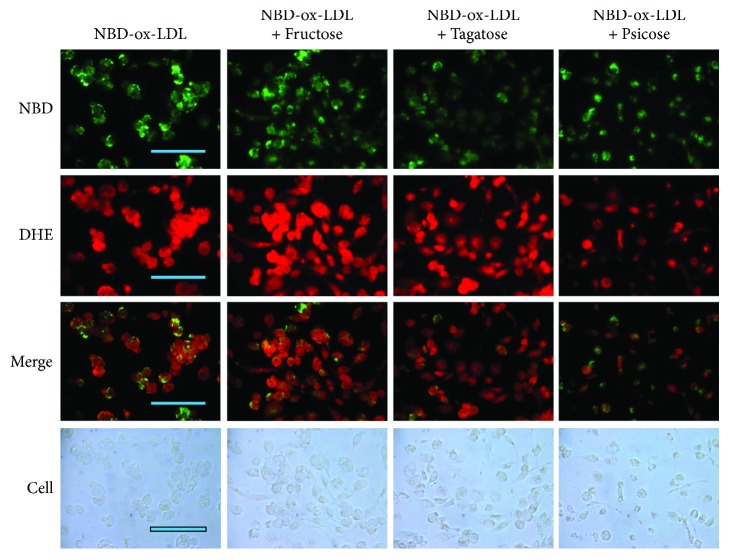
Cellular uptake of oxLDL containing NBD-cholesterol in the presence of each ketohexose (fructose, tagatose, and psicose). PMA-differentiated macrophages were incubated with 50 *μ*L of oxLDL (1 mg/mL), 14 *μ*L of ketohexose alone (175 mM, at final 5 mM), and 436 *μ*L of RPMI1640 media. After incubation with oxLDL and each ketohexose for 48 hrs, cells were washed by PBS (phosphate-buffered saline) and green fluorescence (Ex = 488 nm, Em = 535 nm) intensity was detected. In order to compare production of reactive oxygen species (ROS), red fluorescence image (Ex = 588 nm, Em = 605 nm) was obtained after dihydroethidium (DHE) staining. Cell images were observed and photographed using a Nikon Eclipse TE2000 microscope (Tokyo, Japan). The bar in the photo indicates 100 *μ*m.

**Figure 5:**
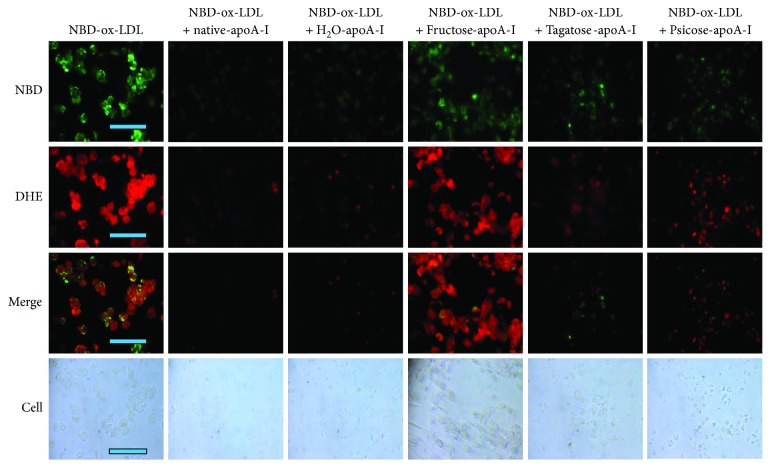
Cellular uptake of oxLDL in the presence of rHDL-containing ketohexose-treated apoA-I and NBD-cholesterol. PMA-differentiated macrophages were incubated with 50 *μ*L of oxLDL (1 mg/mL), 50 *μ*L of each rHDL (0.7 mg/mL, at final 2 *μ*M), and 400 *μ*L of RPMI1640 media. After incubation with oxLDL and each rHDL for 48 hrs, cells were washed by PBS (phosphate-buffered saline) and green fluorescence (Ex = 488 nm, Em = 535 nm) intensity was detected. In order to compare production of reactive oxygen species (ROS), red fluorescence image (Ex = 588 nm, Em = 605 nm) was obtained after dihydroethidium (DHE) staining. Cell images were observed and photographed using a Nikon Eclipse TE2000 microscope (Tokyo, Japan). The bar in the photo indicates 100 *μ*m.

In the presence of ketohexose (final 5 mM) and oxLDL, fructose treatment caused the most severe extent of uptake of oxLDL (green fluorescence) and increased the level of ROS (red fluorescence) as shown in Figure 4. Although oxLDL was slightly less uptaken by fructose than oxLDL alone treatment, production of ROS was more elevated in fructose treatment. These results suggest that basal level of fructose, even in final 5 mM, could enhance more atherogenic process via foam cell formation. However, treatment with tagatose and psicose showed much lesser uptake of oxLDL (green) and smaller ROS production (red). Similarly, the treatment of f-A-I caused the increased uptake of oxLDL and ROS production, while t-A-I- and p-A-I-treated THP-1 cell showed much less oxLDL uptake and ROS level (Figure 5). Taken together, the staining revealed that fructose or f-A-I treatment caused the highest production of ROS level, an indicator of oxidative stress in the cell.

### 3.5. Western Blotting

**Figure 6:**
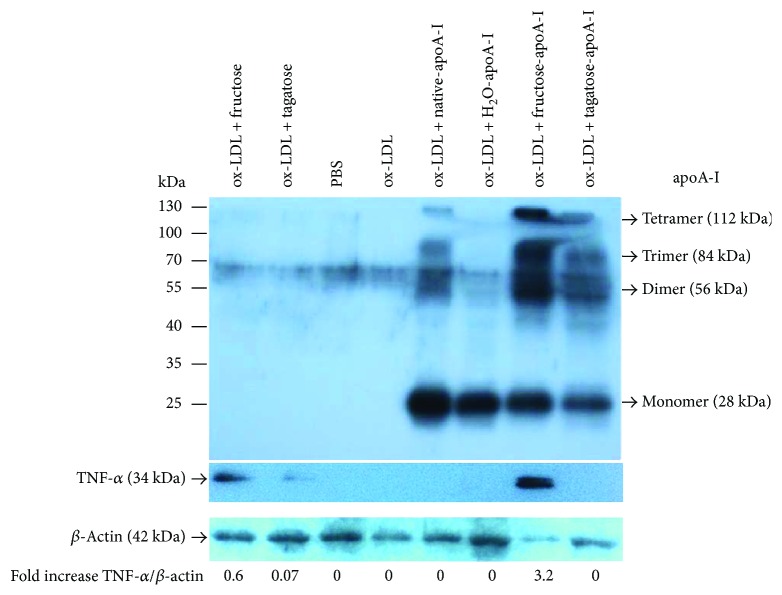
Western blot analysis. An equal amount of protein (13 *μ*g per lane) was loaded on 15% SDS-PAGE. ApoA-I (ab7613; Abcam), tumor necrosis factor (TNF)-*α* (sc52746; Santa cruz biotechnology), and *β*-actin antibodies (ab8229; Abcam) were used.

From immunodetection with apoA-I antibody, under the presence of 2 *μ*M of protein, f-A-I-treated cell showed the strongest multimer band of apoA-I in the lysate of macrophage, while t-A-I-treated cell showed a less multimeric band of apoA-I as similar as native apoA-I (Figure 6). The ApoA-I band was not detected in ketohexose alone (final 5 mM) treated cell as control (lane 1–4, Figure 6), suggesting there was no detectable apoA-I in the macrophage. Interestingly, TNF-alpha band appeared only in fructose alone treated and f-A-I-treated models.

### 3.6. Microinjection of Ketohexose into Zebrafish

**Figure 7:**
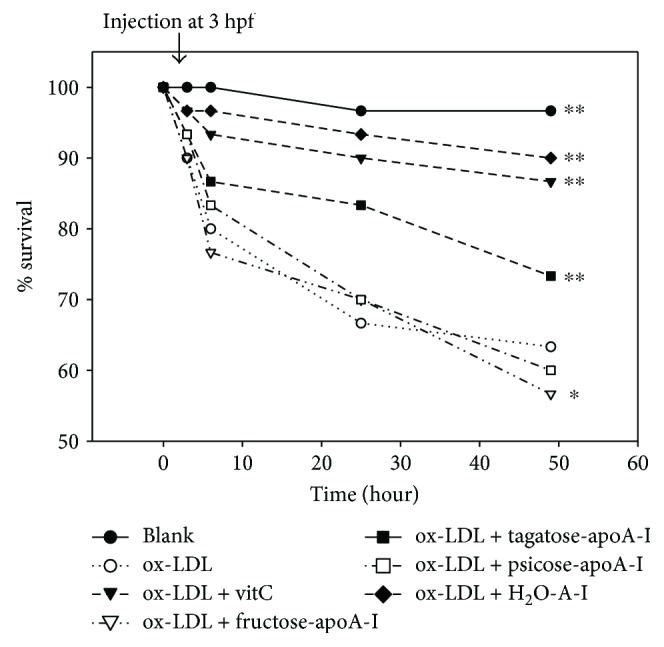
Survival curve of zebrafish embryos after co-injection of oxLDL (13 ng of protein) plus vitamin C (final concentration, 1 *μ*M) or each ketohexoses apo-A-1 during 48 hr incubation post-injection. ^∗^ *P* < 0.05 vs oxLDL; ^∗∗^ *P* < 0.01 vs oxLDL.

**Figure 8:**
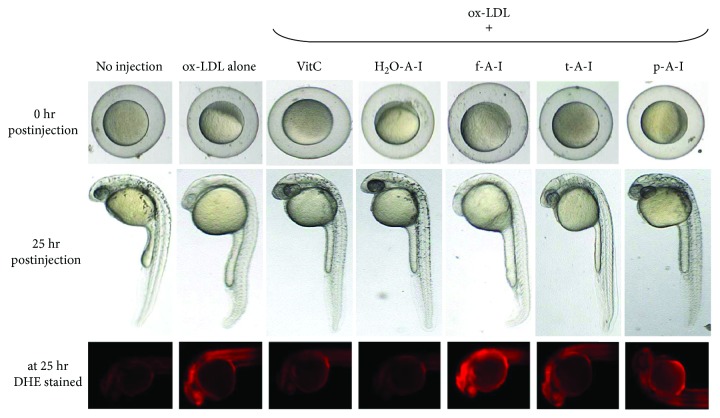
Change in embryo stage after coinjection of oxLDL and ketohexose-treated apoA-I at 0 and 25 hr postinjection. In order to compare production of reactive oxygen species, red fluorescence image (Ex = 588 nm, Em = 605 nm) of zebrafish embryo was obtained using a Nikon Eclipse TE2000 microscope (Tokyo, Japan) after dihydroethidium (DHE) staining as described in the text. f-A-I: fructose-treated apoA-I; t-A-I: tagatose-treated apoA-I; p-A-I: psicose-treated apoA-I; H_2_O-A-I: H_2_O-treated apoA-I.

Microinjection of oxLDL (13 ng of protein) alone into zebrafish embryo resulted in the highest embryo death with around 63% survival as shown in Figure 7, suggesting that oxLDL could cause acute inflammatory death. Under the presence of oxLDL, f-A-I-injected embryo showed the lowest survivability, indicating that the existence of f-A-I made more exacerbation of the inflammatory death. Surprisingly, t-A-I-injected embryo showed higher survivability than oxLDL alone control, while coinjection of Vit-C resulted in the highest survival. At 24 hr postinjection, f-A-I-injected embryo showed the slowest embryo development speed as shown in Figure 8, as well as oxLDL alone, injected embryo. oxLDL alone was treated as a reference category to perform the statistical analysis.

### 3.7. Consumption of Tagatose Caused Hypotriglyceridemia in Zebrafish

After 3 weeks feeding of HCD with or without the ketohexose (50% wt/wt), fructose-administered group (HCHF) and tagatose-administered group (HCHT) showed higher than 90% survival similar with HCD control group, suggesting that excess feeding of ketohexose (50% wt/wt) was well tolerated. In the serum profile, HCD-alone-administered group showed an increment of TC and TG which was found to be significant when the HCD data was compared to the normal. Fructose-fed group showed 15% decrease of serum TC, while tagatose-administered group showed the similar level of serum TC compared with HCD group. However, the serum TG level was 14% more increased in fructose group, while tagatose-fed group showed 30% decrease of serum TG compared with HCD control. The fructose-fed group showed 1.6-fold higher serum TG level than the tagatose-fed group. Fructose-fed group also showed the highest serum glucose level, 43% more increased than HCD group. However, tagatose-fed group showed smaller glucose level than the fructose-fed group and these results were statistically significant. Serum GOT was also elevated in HCD group (Table 2), suggesting that hyperlipidemia is associated with acute hepatic inflammation. The fructose-fed group showed an increment of 22% in the serum GOT level; nonetheless, tagatose-fed group showed 15% more decreased than that of HCD group.

Serum CETP activity was more enhanced by HCD consumption than ND group, 48 ± 4% and 37 ± 1% of CE-transfer, respectively. However, HCHT group showed significantly decreased CETP activity up to 42% CE-transfer, while HCHF group showed similar CETP activity as HCD group. These results suggest that HC consumption can cause enhancement of CETP activity as our previous report and tagatose consumption could diminish the activity.

### 3.8. Histologic Analysis

**Figure 9:**
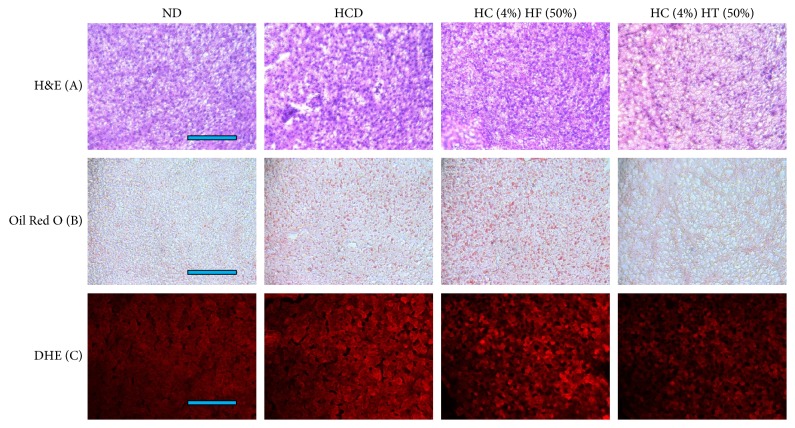
Representative micrographs for histological assessments of hepatic microsections. The bar in the photo indicates 100 *μ*m. (a) Infiltrated inflammatory cells were visualized by hematoxylin and eosin staining. (b) Extent of fatty liver change was measured by Oil Red O staining. (c) Production of reactive oxygen species were determined by DHE staining.

From H & E staining, HC consumption caused more infiltration of inflammatory cells in hepatic microsections as shown in Figure 9. However, HCHT-consumed group showed much less infiltration of the cells, while HCHF group showed a similar level of the infiltration. Oil Red O staining revealed that HC group showed a remarkable increase of red intensity compared with normal group, suggesting cholesterol consumption caused fatty liver change. HCHF group showed stronger red intensity than HC group, indicating that fructose consumption accelerates the fatty liver change. However, surprisingly, HCHT group showed almost no Oil Red O-stained area as similar level as the normal group. These results suggest that tagatose consumption can ameliorate inflammatory aggravation and fatty liver change caused by cholesterol consumption.

## 4. Discussion

Generally, glycation is associated with structural modification and functional loss of serum proteins, especially hemoglobin and apolipoproteins. Our research group and several others have reported that glycation of apolipoprotein is involved in the critical process of diabetes, atherosclerosis, and senescence [11, 14]. Previous reports suggested that among ketohexoses, tagatose tastes like sucrose and is useful as a low-calorie sweetener. Tagatose has lower glycemic index compared to the other sweeteners and ketohexoses. It has been reported that a low glycemic index diet over many years lowers the risk of developing type 2 diabetes, CVD, and other metabolic risks [32]. Moreover, tagatose appears to have antihyperglycemic effect and obesity control drug by reducing the blood glucose among normal and prediabetic participants possibly through inhibiting intestinal disaccharidases and glucose transport. Tagatose decreases the level of postprandial glucose and modulates the insulin response through inhibition of glycogenolysis in the liver [33, 34].

Interestingly, t-A-I showed highest formation of multimer than f-A-I and p-A-I (Figure 1(b)), suggesting that tagatose could modify more BS^3^-crosslinking site. However, there was no notable difference in the crosslinking efficiency between ketohexose-treated apoA-I in rHDL state, although all ketohexose-treated apoA-I showed much more dimerization than control (H_2_O treated).

The multimerization ability and phospholipid-binding ability of t-A-I might suggest that critical amino acid is modified by the tagatose treatment. However, the modification caused less damage of beneficial functions of apoA-I and rHDL.

More uptake of cholesterol from oxLDL into macrophage is associated with increased production of ROS (Figure 4). Especially in the presence of fructose, the extent of cholesterol uptake was similar compared with oxLDL alone, however, the production of ROS was stronger than oxLDL alone. Among ketohexose-treated apoA-I, f-A-I showed the highest uptake of NBD-oxLDL and production of ROS, suggesting that coincidence of oxLDL phagocytosis and oxidative stress (Figure 5). As shown in Figure 6, immunodetection with TNF-*α* antibody revealed that fructose-treated (lane 1) or f-A-I-treated (lane 7) cell lysate showed distinct band, while tagatose or t-A-I-treated cell did not.

The proinflammatory properties of fructose appeared again in the zebrafish embryo with the highest mortality and the slowest development speed as similar as an oxLDL alone injection. These results make a good agreement with the previous report that embryos injected with oxLDL alone had a more attenuated developmental speed than native LDL-injected embryos [35]. Furthermore, coinjection of fructosylated apoA-I and oxLDL exacerbated the embryo death with the slowest developmental speed, while native apoA-I showed a protective effect. While p-A-I showed more production of ROS in the macrophage and less protective effect against the oxLDL mediated embryo death, however, t-A-I showed more protective effect both in the macrophage and the embryo.

After 3 weeks feeding, HCD consumption caused infiltration of inflammatory cells and fatty liver change based on our previous finding in mouse model and recent reports draw attention on the deleterious effect of HCD consumption that caused severe steatohepatitis along with early liver fibrosis [36–38]. As shown in Table 2, tagatose-fed group showed mild weight loss effect compared with HCD control group. In human study, Donner et al. showed that 12 months of oral administration of tagatose resulted in weight loss effect, from 109 ± 14.7 kg to 105.3 ± 14.4 kg, along with a reduction in glycohemoglobin [16]. Although the study was based on a small number of subjects (*n* = 8), the weight loss effect made a good agreement with the current result. In a similar study on hypercholesterolemic mice, Police et al. revealed that in comparison to sucrose, an equal amount of tagatose did not elevate the risk of hyperglycemia, hyperlipidemia, and resulted in a lesser extent of hypercholesterolemia and atherosclerosis [39].

Surprisingly, fructose-treated group (HCHF) showed reduction in body weight and serum TC level compared with HCD group. More interestingly, the HCHF group revealed lower body weight than ND group, suggesting that excess dosage of fructose could result in severe weight loss. However, the group showed severe inflammation in hepatic tissue (Figure 9). This acute weight loss might be connected with fatty liver change and hepatosteatosis. The HCHF group showed the highest serum TG and inflammatory level. Similar reports of hepatic lipid accumulation, inflammation, and oxidative stress were shown by Sapp et al. and Jaiswal et al. after fructose treatment in larval zebrafish and L6 skeletal muscle cells [40, 41]. Our results make a good agreement that f-A-I-treated cell showed the highest elevation of TNF-*α*.

## 5. Conclusions

To conclude, tagatose may modify several functions of apoA-I such as phospholipid-binding ability and multimerization ability. However, the antioxidant and anti-inflammatory activity of apoA-I was not impaired in the macrophage and zebrafish embryo model. In hypercholesterolemic zebrafish model, tagatose-consumed group showed a reduction in serum TG, GOT, and CETP activity.

## Figures and Tables

**Table 1:** Synthesis and characterization of rHDL containing each ketohexose (fructose, tagatose, and psicose).

rHDL	Molar composition	WMF (nm)	Size^a^ (Å)	Number of apoA-I/particle^b^	*α*-Helicity (%)
Native-A-I	95 : 5 : 1	337 ± 0.1 (344 ± 0.5)^c^	109–94		54.4 (32.6)^c^
H_2_O-A-I	95 : 5 : 1	335 ± 0.5 (344 ± 0.5)	109–94	2, 3, 4	82.6 (51.4)
f-A-I	95 : 5 : 1	342 ± 1.6 (344 ± 0.5)	109–94	2, 3, 4, 5	38.4 (38)
t-A-I	95 : 5 : 1	335 ± 1.2 (344 ± 0.5)	93	2, 3, 4, 5	34.7 (15.4)
p-A-I	95 : 5 : 1	335 ± 0.4 (344 ± 0.5)	93	2, 3, 4, 5	35 (25.4)

^a^Determined from 8% to 25% native-gradient gel electrophoresis with densitometric scanning analysis. ^b^Determined from BS_3_-crosslinking and 8–25% SDS–PAGGE. ^c^The numbers in the parentheses indicate the proteins in the lipid-free state. H_2_O-A-I: H_2_O-treated apoA-I; f-A-I: fructose-treated apoA-I; t-A-I: tagatose-treated apoA-I; p-A-I: psicose-treated apoA-I.

**Table 2:** Serum profile of zebrafish after consumption of high cholesterol diet and ketohexose for 3 weeks.

	ND^1^ (*n* = 70)	HCD^2^ (*n* = 70)	HCHF^3^ (*n* = 70)	HCHT^4^ (*n* = 70)
Weight (mg)/height (mm)	9.2 ± 1.6	9.9 ± 2.0^∗^	8.9 ± 1.5	9.3 ± 1.9
Total cholesterol (mg/dL)	108 ± 5	385 ± 15^∗∗^	330 ± 2^∗∗^	407 ± 34^∗∗^
Tricylglyceride (mg/dL)	232 ± 10	271 ± 51^∗∗^	311 ± 31^∗∗^	194 ± 13^∗∗^
Glucose (mg/dL)	54 ± 16	73 ± 18^∗∗^	105 ± 25^∗∗^	83 ± 26^∗∗^
GOT (Karmen/mL)	178 ± 4	221 ± 31^∗∗^	271 ± 27^∗∗^	191 ± 17^∗∗^
CETP activity (% CE-transfer/4 h)	37 ± 1	48 ± 6^∗∗^	49 ± 1^∗∗^	42 ± 0^∗∗^

^1^ND: normal diet, Tetrabit®: Tetrabit (47.5% crude protein, 6.5% crude fat, 2.0% crude fiber, 10.5% crude ash, containing vitamin A [29,770 IU/kg], vitamin D3 [1860 IU/kg], vitamin E [200 mg/kg], and vitamin C [137 mg/kg]). ^2^HC: high cholesterol (4% cholesterol in ND, wt/wt). ^3^HCHF: high cholesterol and high fructose (50% fructose in HCD, wt/wt). ^4^HCHT: high cholesterol and high tagatose (50% tagatose in HCD, wt/wt). Data were analyzed by ANOVA Bonferroni *t*-test. Levels of significance were represented in the form of ^∗∗^ *P* < 0.001, ^∗^ *P* < 0.05 when compared with initial (ND) group. TC: total cholesterol; TG: triglycerides; GOT: glutamate oxaloacetate transaminase; CETP: cholesteryl ester transfer protein.

## Supplementary Materials

Supplementary Materials Supplementary Figure 1: electrophoretic patterns of the glycated apoA-I in the lipid-free (A) and lipid-bound states on native gel electrophoresis. Supplementary Figure 2: circular dichroism spectra with ketohexose-treated apoA-I in lipid-free (A) and lipid-bound states (B). f-A-I: fructose-treated apoA-I; t-A-I: tagatose-treated apoA-I; p-A-I: psicose-treated apoA-I.
