# Supplementary material for: The Ability of Different Ketohexoses to Alter Apo-A-I Structure and Function In Vitro and to Induce Hepatosteatosis, Oxidative Stress, and Impaired Plasma Lipid Profile in Hyperlipidemic Zebrafish
Source: Oxid Med Cell Longev. 2018 May 21;2018:3124364. doi: 10.1155/2018/3124364 (PMC5987316; doi:10.1155/2018/3124364)
Supplement: Supplementary Materials — Supplementary Figure 1: electrophoretic patterns of the glycated apoA-I in the lipid-free (A) and lipid-bound states on native gel electrophoresis. Supplementary Figure 2: circular dichroism spectra with ketohexose-treated apoA-I in lipid-free (A) and lipid-bound states (B). f-A-I: fructose-treated apoA-I; t-A-I: tagatose-treated apoA-I; p-A-I: psicose-treated apoA-I. [file 3124364.f1.pptx]

## Slide 1
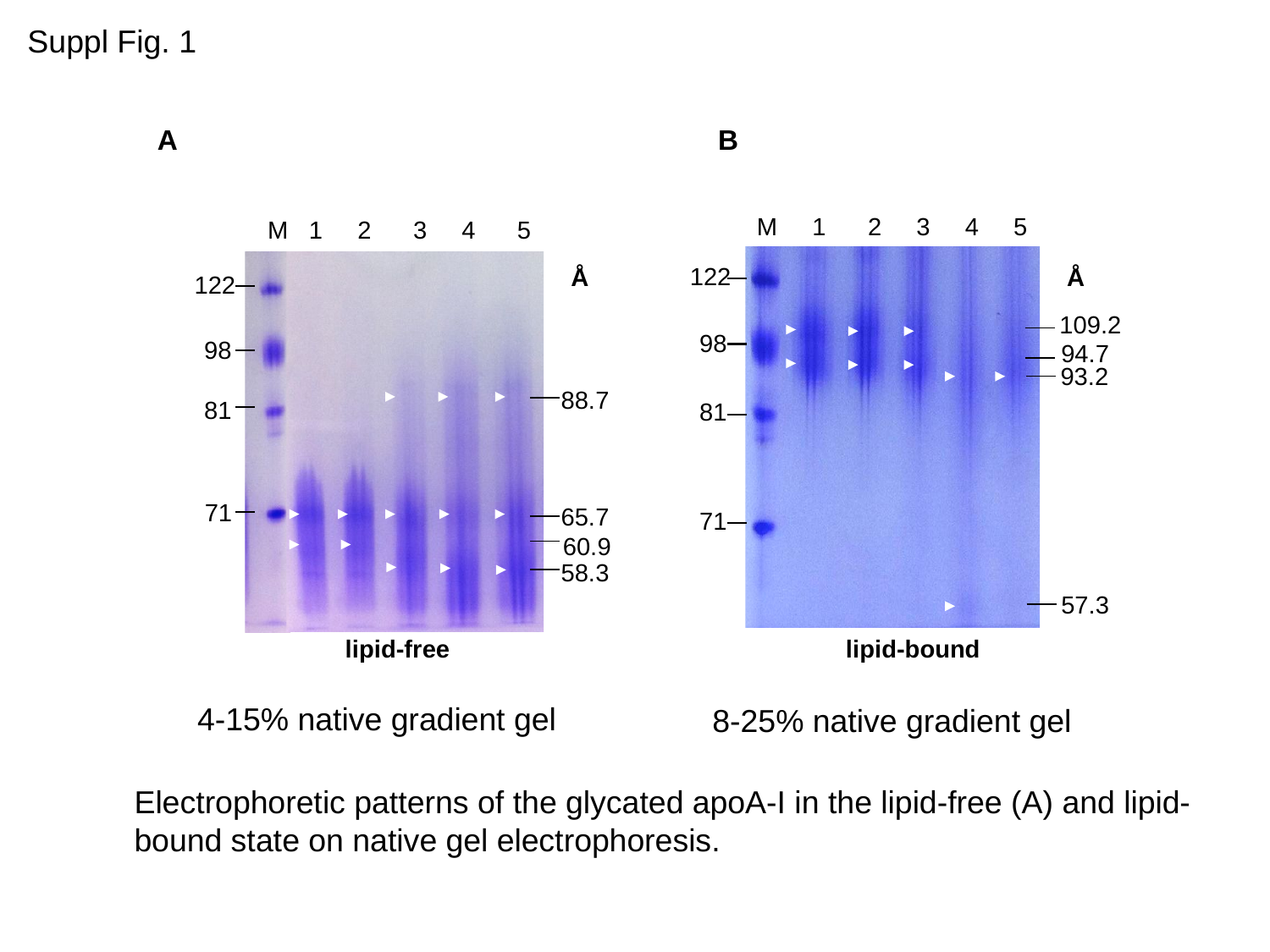

Suppl Fig. 1
A
B
M 1 2 3 4 5
M 1 2 3 4 5
122
Å
Å
122
109.2
98
98
94.7
93.2
88.7
81
81
71
65.7
71
60.9
58.3
57.3
lipid-free
lipid-bound
4-15% native gradient gel
8-25% native gradient gel
Electrophoretic patterns of the glycated apoA-I in the lipid-free (A) and lipid-bound state on native gel electrophoresis.

## Slide 2
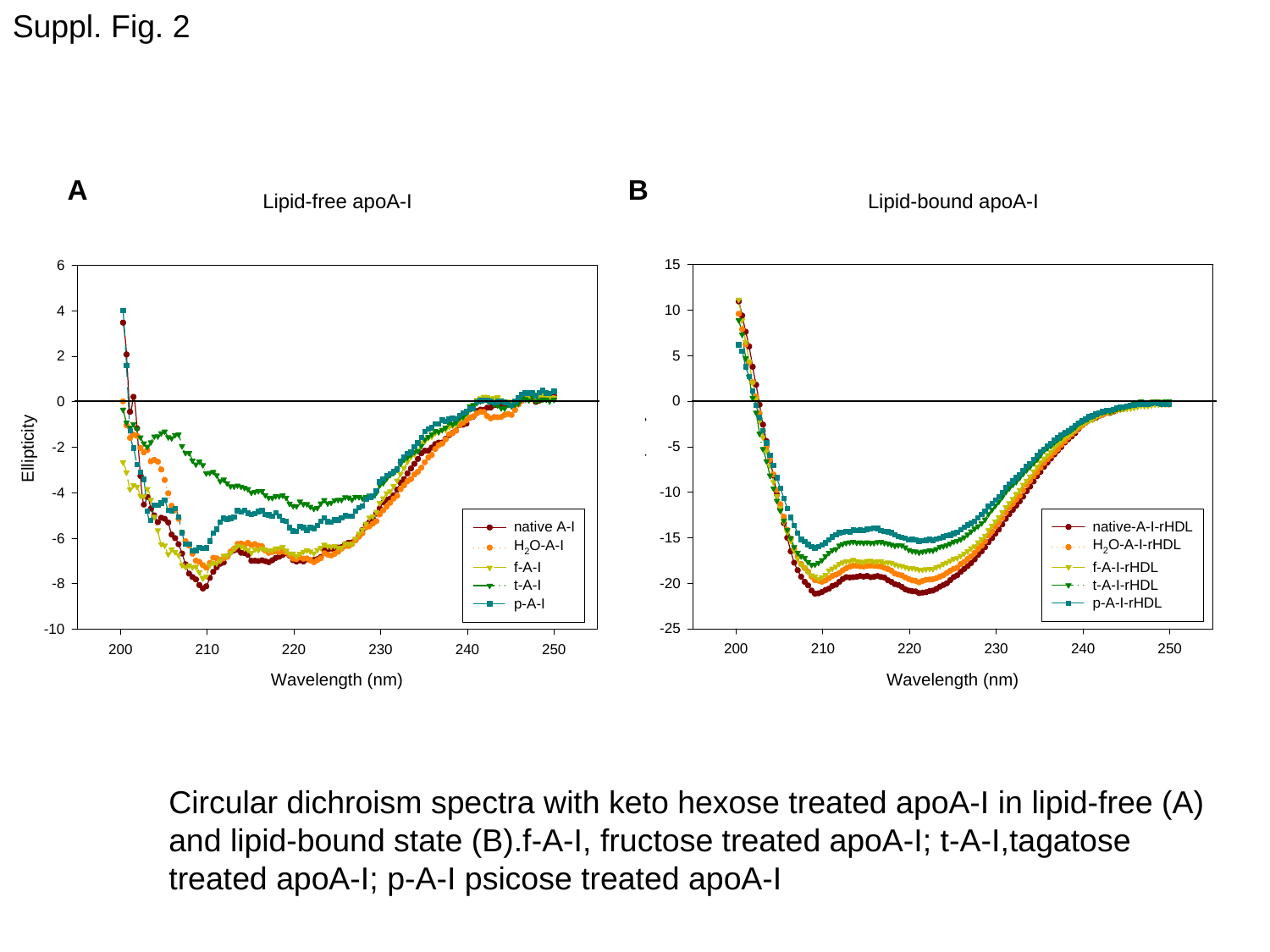

Suppl. Fig. 2
A
B
Circular dichroism spectra with keto hexose treated apoA-I in lipid-free (A) and lipid-bound state (B).f-A-I, fructose treated apoA-I; t-A-I,tagatose treated apoA-I; p-A-I psicose treated apoA-I
